# The Place of Appeals in Hospital Support

**Published:** 1914-12-05

**Authors:** 


### SOME CARDINAL POINTS OF VOLUNTARY FINANCE.

### The Place of Appeals in Hospital Support.

BY A HOSPITAL SUPERINTENDENT.

It is delightful to read the comment in THE HOSPITAL of November 21 on the five cardinal points of an appeal, as though the appeal constituted the principal, or perhaps the sole, means of obtaining money for the voluntary hospitals.* The appeal, after all, is but one of the forces by which the income of the voluntary hospitals is raised. A successful hospital obtains its support by reason of the appeal to the public, which reaches them through practically every branch of the hospital's constitution. One has only to look round and study those hospitals which meet with the greatest support, and which can record the longest list of legacies, to see that the appeal itself is but a very minor factor in the success that has been attained.

Those hospitals are fortunate which have upon the honorary medical staff men who devote a large portion of their lives to the interests of the hospitals they serve, men who more often than not are *alumni* of the institution that boasts them as physicians or surgeons, men who in their daily practice mention the name of their hospital, record incidents of its work, and, without actually asking for money, place ever before their patients and friends glowing accounts of the work done in the wards, and the general efficiency of the institution.

This probably constitutes the greatest appeal that any hospital knows. It cannot be worked from the office, it cannot be stimulated by the presence of a subscription box upon the consulting-room table, it cannot be augmented by reminders—it must be in the spirit of the men who love their *Alma Mater*; and if it is there, and they are of the right sort, the hospital they serve benefits immensely. A legacy is more often left to the hospital in consequence of something the doctor has said than of all the advertisements and appeals which the most active and energetic secretary can make.

This probably constitutes the first plank in the financial success of any voluntary hospital, and next to this comes the influence of the board of management. There are those hospitals so fortunate as to have men serving regularly upon the board, devoting themselves to the one institution, who lose no opportunity of bringing people to see the institution, of talking about it to their friends, and of always letting it be seen that whatever may be said of any rival charity, the one they serve is the one most worthy of support.

### THE NURSING DEPARTMENT'S FINANCIAL OPPORTUNITY.

No less important is the nursing department. Glance at those hospitals which have succeeded, and it will be observed, so close is the understanding between the nursing department and the board, that every effort is made by the former to carry on the training of nurses in such a way that at any rate some portion of it will bring good supporters and high interest to the particular charity. We see this in those hospitals which have risen to the occasion since the war first commenced. It is all very well for the nursing arrangements to be cut and dried, for three or four years' training to be insisted upon, and for a particular class only to be admitted; but there are times like the present when golden stores are accumulating for the hospitals which have relaxed their rules to meet the wish of the public. All of us who know the hospital world can put our finger on the particular institution where nurses (save the word!) are admitted to the wards for any period whatever. "Lady This" and the "Hon. Miss That" can learn nursing by attending for an hour or an hour and a half on two days in the week, or they may go in for the whole week or fortnight, or every second Monday, or what not. They do not do much harm, especially if they are put in a ward with an experienced sister; but they bring untold wealth to the hospital to which they go. Probably for the rest of their lives they will tell everyone they meet of the wonders of that particular hospital in which they spent those eventful days. Often as not they are rich, or have rich relations who can leave money to the hospital, and wise is the board of management, and wise the matron's department, who can see things in this light and can thus aid in bringing money to the charity for the better treatment of the sick poor in the future.

### THE POTENTIAL VALUE OF WORTHLESS GIFTS.

In little things so much can be done of an appeal nature. I know the secretary of a hospital who always says that he cannot stand people who send old newspapers and books, and that for such gifts he never sends a letter of thanks. Anything more absurd cannot be imagined. A contribution is often the outcome of a petty gift either from the donor or from the donor's rich friend. Quite lately a hospital secretary was asked to go to a neighbouring house. When he arrived there the lady of the house said that she was interested in the hospital and would like to make it a present. She produced a wicker basket practically worn out, and an old surgical appliance which had belonged to a dead and gone ancestor. This was duly accepted and a letter of appreciation written from the office afterwards. Other insignificant gifts followed, then the endowment of a bed. The letter of appeal constitutes but a small factor in the financing of our voluntary hospitals, but it is a factor which must never be neglected, and which bears fruit when it falls upon soil already tilled and prepared for it.

Good common sense, friendly notes, no printed matter, prompt replies, and acknowledgments of

* This was not our suggestion.—ED.

the most insignificant trifles. There is no one factor, the whole charity from the president downward must work to augment the income, to meet the wishes of the public, which at times is most unreasonable, and without sacrificing principles, to fall in with little wishes and little desires, and generally to take all those, rich or poor, who are in touch with you into your confidence.

The Possibilities of the Junior Staff.

A word in regard to the junior staff. It seems impossible to those who have worked all their lives as laymen in hospitals that the men who act as house surgeons should ever develop into the honorary medical staff. No one who knows the medical profession intimately can be blind to the integrity, the generosity, the ability, and knowledge of the world displayed by those who have attained high position in the medical profession. On the other hand, he cannot but be also alive to the fact that there are few men so little worldly-wise or so unversed in the ways of the world as the junior resident medical officer. The houseman who is endowed with tact and a gentle manner can, and frequently does, bring much support to the hospital. A casualty officer with these attributes is invaluable. We all know the garrulous nature of the poor, especially in regard to their ailments, and their reception in the casualty room is told to every friend, to their employer, and to their patron. However unreasonable it may seem that persons of education should be moved by stories told by out-patients and casualty patients, the fact remains that they are influenced, and that they give or withhold their benefactions to the charity according to the story which they hear. Unhappily the junior medical officer is young for his age, late in developing, and it would perhaps be unreasonable to expect that he should realise his indebtedness to the charity which is giving him education and experience. It is unfortunate that in a large number of cases he is almost the first person with whom the general public come in contact.

The Hall Porter.

Few have ever thought of the influence of the hall porter. A rough or uncouth manner, discourtesy, or slovenliness have turned away many from the doors of the hospitals in the past. His is a difficult billet, and it is not to be expected that he can differentiate between one person and another in certain difficult circumstances. So it may come about that many a person entering the hospital in good will leaves it never to approach it again, and perhaps never to have any more to do with a medical charity. Every hospital can take guarantees against such risks by careful selection, intimate observation, the payment of good wages, and a stern insistence upon constant smartness and courtesy on the part of their hall porter.

